# (5-Bromo-2-hy­droxy­phen­yl)(4-propyl­cyclo­hex­yl)methanone

**DOI:** 10.1107/S1600536812011634

**Published:** 2012-03-24

**Authors:** Kang Meng, Miao Cao, Zhuo-Ling An, Jie Zhang, Li-Hong Liu

**Affiliations:** a Pharmacy Department of The Second Artillery General Hospital, Beijing 100088, People’s Republic of China

## Abstract

In the title compound, C_16_H_21_BrO_2_, the cyclo­hexane ring adopts a chair conformation. The hy­droxy and carbonyl groups are involved in an intra­molecular O—H⋯O hydrogen bond. In the crystal, weak C—H⋯O inter­actions link the mol­ecules into zigzag chains along [010].

## Related literature

For details of the biological activity of SGLT2 inhibitors, see: Meng *et al.* (2008 ▶); Gao *et al.* (2010 ▶); Shao *et al.* (2011 ▶). For related structures, see: Robinson *et al.* (2002 ▶); Wang *et al.* (2011 ▶).
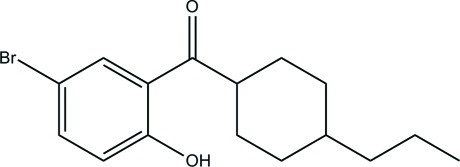


## Experimental

### 

#### Crystal data

C_16_H_21_BrO_2_
*M* *_r_* = 325.24
Monoclinic, 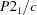

*a* = 14.5826 (12) Å
*b* = 8.5467 (8) Å
*c* = 12.6369 (10) Å
β = 113.037 (5)°
*V* = 1449.4 (2) Å^3^
*Z* = 4
Mo *K*α radiation
μ = 2.83 mm^−1^
*T* = 113 K
0.20 × 0.18 × 0.12 mm

#### Data collection

Rigaku Saturn 724 CCD area-detector diffractometer
Absorption correction: multi-scan (*CrystalClear-SM Expert*; Rigaku/MSC, 2009 ▶) *T*_min_ = 0.601, *T*_max_ = 0.727
17988 measured reflections
3441 independent reflections
3069 reflections with *I* > 2σ(*I*)
*R*_int_ = 0.035

#### Refinement

*R*[*F* ^2^ > 2σ(*F* ^2^)] = 0.028
*wR*(*F* ^2^) = 0.062
*S* = 1.04
3441 reflections
177 parameters
H atoms treated by a mixture of independent and constrained refinement
Δρ_max_ = 0.81 e Å^−3^
Δρ_min_ = −0.34 e Å^−3^

### 

Data collection: *CrystalClear-SM Expert* (Rigaku/MSC, 2009 ▶); cell refinement: *CrystalClear-SM Expert*; data reduction: *CrystalClear-SM Expert*; program(s) used to solve structure: *SHELXS97* (Sheldrick, 2008 ▶); program(s) used to refine structure: *SHELXL97* (Sheldrick, 2008 ▶); molecular graphics: *SHELXTL* (Sheldrick, 2008 ▶); software used to prepare material for publication: *SHELXTL*.

## Supplementary Material

Crystal structure: contains datablock(s) I, global. DOI: 10.1107/S1600536812011634/cv5261sup1.cif

Structure factors: contains datablock(s) I. DOI: 10.1107/S1600536812011634/cv5261Isup2.hkl

Supplementary material file. DOI: 10.1107/S1600536812011634/cv5261Isup3.cml

Additional supplementary materials:  crystallographic information; 3D view; checkCIF report

## Figures and Tables

**Table 1:** Hydrogen-bond geometry (Å, °)

*D*—H⋯*A*	*D*—H	H⋯*A*	*D*⋯*A*	*D*—H⋯*A*
O1—H1⋯O2	0.81 (2)	1.82 (2)	2.5527 (16)	148 (3)
C3—H3⋯O1^i^	0.95	2.59	3.483 (2)	157

Symmetry code: (i) 
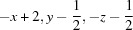
.

**Fig. 1.**
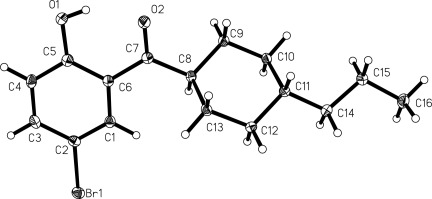
View of the title compound, with displacement ellipsoids drawn at the 40% probability level.

## supplementary crystallographic information

### Comment

SGLT2 inhibitors represent a new class of potential hypoglycemic agents (Meng *et al.*, 2008). During the development of our own cyclohexane-bearing SGLT2 inhibitors (Gao *et al.*, 2010; Shao *et al.*, 2011), the title compound (I) was prepared as a key intermediate.

In (I) (Fig. 1), all bond lengths and angles are normal and in a good agreement with those reported previously for related compounds (Robinson *et al.*, 2002; Wang *et al.*, 2011). The cyclohexane ring (C8—C13) adopts a chair conformation. Weak intermolecular C—H···O interactions (Table 1) link the molecules into zigzag chains in [010].

### Experimental

A dried 100-ml round-bottomed flask was charged with 1.89 g (10 mmol) of *trans*-4-propylcyclohexanecarboxylic acid chloride, 1.87 g (10 mmol) of 4-bromoanisole and 20 ml of dried dichloromethane. The mixture was stirred on an ice-water bath, followed by addition of 1.60 g (12 mmol) of anhydrous aluminium chloride in a portionwise manner. After addition, the reaction mixture was stirred at room temperature for 1 h and at reflux overnight, and poured into 300 ml of ice-water. The mixture thus formed was exacted with three 50-ml portions of dichloromethane, and the combined exacts were washed with saturated brine, dried over sodium sulfate and evaporated on a rotary evaporator to afford the crude title compound. Pure title compound was obtained by column chromatography. Crystals suitable for X-ray diffraction were obtained through slow evaporation of a solution of the pure title compound in dichloromethane/petroleum ether (1/30 by volume).

### Refinement

Hydroxy atom H1 was located on a difference map and isotropically refined. C-boundl H atoms were geometrically positioned [C–H = 0.95–1.00 Å], and included in the final cycles of refinement using a riding model, with *U*_iso_(H) = 1.2*U*_eq_(C) and 1.5*U*_eq_(C) for the methyl H atoms.

### Crystal data

**Table:**

C_16_H_21_BrO_2_	*F*(000) = 672
*M**_r_* = 325.24	*D*_x_ = 1.490 Mg m^−^^3^
Monoclinic, *P*2_1_/*c*	Mo *K*α radiation, λ = 0.71073 Å
Hall symbol: -P 2ybc	Cell parameters from 5072 reflections
*a* = 14.5826 (12) Å	θ = 1.8–27.9°
*b* = 8.5467 (8) Å	µ = 2.83 mm^−^^1^
*c* = 12.6369 (10) Å	*T* = 113 K
β = 113.037 (5)°	Prism, colourless
*V* = 1449.4 (2) Å^3^	0.20 × 0.18 × 0.12 mm
*Z* = 4	

### Data collection

**Table:**

Rigaku Saturn 724 CCD area-detector diffractometer	3441 independent reflections
Radiation source: rotating anode	3069 reflections with *I* > 2σ(*I*)
Multilayer monochromator	*R*_int_ = 0.035
Detector resolution: 14.22 pixels mm^-1^	θ_max_ = 27.9°, θ_min_ = 2.8°
ω and φ scans	*h* = −19→19
Absorption correction: multi-scan (*CrystalClear-SM Expert*; Rigaku/MSC, 2009)	*k* = −11→11
*T*_min_ = 0.601, *T*_max_ = 0.727	*l* = −16→16
17988 measured reflections	

### Refinement

**Table:**

Refinement on *F*^2^	Primary atom site location: structure-invariant direct methods
Least-squares matrix: full	Secondary atom site location: difference Fourier map
*R*[*F*^2^ > 2σ(*F*^2^)] = 0.028	Hydrogen site location: inferred from neighbouring sites
*wR*(*F*^2^) = 0.062	H atoms treated by a mixture of independent and constrained refinement
*S* = 1.04	*w* = 1/[σ^2^(*F*_o_^2^) + (0.0334*P*)^2^ + 0.0947*P*] where *P* = (*F*_o_^2^ + 2*F*_c_^2^)/3
3441 reflections	(Δ/σ)_max_ = 0.002
177 parameters	Δρ_max_ = 0.81 e Å^−^^3^
0 restraints	Δρ_min_ = −0.34 e Å^−^^3^

### Special details

**Table:**

Geometry. All e.s.d.'s (except the e.s.d. in the dihedral angle between two l.s. planes) are estimated using the full covariance matrix. The cell e.s.d.'s are taken into account individually in the estimation of e.s.d.'s in distances, angles and torsion angles; correlations between e.s.d.'s in cell parameters are only used when they are defined by crystal symmetry. An approximate (isotropic) treatment of cell e.s.d.'s is used for estimating e.s.d.'s involving l.s. planes.
Refinement. Refinement of *F*^2^ against ALL reflections. The weighted *R*-factor *wR* and goodness of fit *S* are based on *F*^2^, conventional *R*-factors *R* are based on *F*, with *F* set to zero for negative *F*^2^. The threshold expression of *F*^2^ > σ(*F*^2^) is used only for calculating *R*-factors(gt) *etc*. and is not relevant to the choice of reflections for refinement. *R*-factors based on *F*^2^ are statistically about twice as large as those based on *F*, and *R*- factors based on ALL data will be even larger.

### Fractional atomic coordinates and isotropic or equivalent isotropic displacement parameters (Å^2^)

**Table:**

	*x*	*y*	*z*	*U*_iso_*/*U*_eq_	
Br1	1.101943 (12)	0.65152 (2)	0.057741 (15)	0.02149 (7)	
O1	0.80421 (9)	1.03963 (14)	−0.31892 (10)	0.0200 (3)	
H1	0.759 (2)	1.066 (3)	−0.301 (2)	0.058 (8)*	
O2	0.69906 (9)	1.05226 (14)	−0.19829 (10)	0.0202 (3)	
C1	0.92537 (13)	0.83440 (18)	−0.04336 (14)	0.0169 (3)	
H1A	0.9181	0.8160	0.0271	0.020*	
C2	1.00541 (12)	0.77214 (19)	−0.06037 (14)	0.0174 (3)	
C3	1.01921 (13)	0.8002 (2)	−0.16215 (15)	0.0197 (4)	
H3	1.0747	0.7565	−0.1733	0.024*	
C4	0.95127 (13)	0.8920 (2)	−0.24620 (15)	0.0193 (4)	
H4	0.9610	0.9135	−0.3148	0.023*	
C5	0.86871 (13)	0.95348 (19)	−0.23174 (14)	0.0170 (3)	
C6	0.85425 (12)	0.92496 (19)	−0.12913 (14)	0.0153 (3)	
C7	0.76554 (12)	0.99105 (19)	−0.11519 (14)	0.0157 (3)	
C8	0.75600 (12)	0.98403 (19)	−0.00005 (13)	0.0153 (3)	
H8	0.8240	0.9946	0.0623	0.018*	
C9	0.69103 (13)	1.11868 (19)	0.01104 (14)	0.0174 (4)	
H9A	0.7221	1.2196	0.0053	0.021*	
H9B	0.6247	1.1133	−0.0530	0.021*	
C10	0.67844 (13)	1.11146 (19)	0.12561 (14)	0.0169 (4)	
H10A	0.7440	1.1282	0.1893	0.020*	
H10B	0.6335	1.1968	0.1283	0.020*	
C11	0.63570 (12)	0.95477 (18)	0.14288 (13)	0.0154 (3)	
H11	0.5675	0.9435	0.0812	0.018*	
C12	0.70000 (13)	0.82152 (19)	0.12971 (14)	0.0174 (4)	
H12A	0.6697	0.7205	0.1368	0.021*	
H12B	0.7668	0.8276	0.1928	0.021*	
C13	0.71144 (13)	0.82583 (19)	0.01480 (14)	0.0164 (3)	
H13A	0.6456	0.8109	−0.0487	0.020*	
H13B	0.7556	0.7396	0.0116	0.020*	
C14	0.62659 (13)	0.9434 (2)	0.25955 (14)	0.0183 (4)	
H14A	0.5998	0.8389	0.2656	0.022*	
H14B	0.6942	0.9509	0.3211	0.022*	
C15	0.56089 (13)	1.0669 (2)	0.28140 (15)	0.0202 (4)	
H15A	0.4955	1.0692	0.2156	0.024*	
H15B	0.5923	1.1709	0.2869	0.024*	
C16	0.54478 (14)	1.0347 (2)	0.39195 (15)	0.0247 (4)	
H16A	0.5087	0.9359	0.3843	0.037*	
H16B	0.5059	1.1201	0.4056	0.037*	
H16C	0.6095	1.0276	0.4568	0.037*	

### Atomic displacement parameters (Å^2^)

**Table:**

	*U*^11^	*U*^22^	*U*^33^	*U*^12^	*U*^13^	*U*^23^
Br1	0.01672 (10)	0.02144 (10)	0.02637 (11)	0.00224 (7)	0.00849 (8)	0.00249 (7)
O1	0.0228 (7)	0.0202 (6)	0.0201 (6)	0.0024 (5)	0.0115 (6)	0.0036 (5)
O2	0.0184 (6)	0.0240 (6)	0.0187 (6)	0.0029 (5)	0.0077 (5)	0.0018 (5)
C1	0.0178 (8)	0.0165 (8)	0.0184 (8)	−0.0028 (7)	0.0094 (7)	−0.0016 (7)
C2	0.0158 (8)	0.0140 (8)	0.0215 (9)	−0.0014 (7)	0.0063 (7)	−0.0009 (7)
C3	0.0195 (9)	0.0169 (8)	0.0267 (9)	−0.0027 (7)	0.0135 (8)	−0.0054 (7)
C4	0.0225 (9)	0.0189 (8)	0.0213 (9)	−0.0041 (7)	0.0139 (8)	−0.0025 (7)
C5	0.0202 (9)	0.0138 (8)	0.0176 (8)	−0.0040 (7)	0.0079 (7)	−0.0033 (7)
C6	0.0168 (8)	0.0129 (8)	0.0173 (8)	−0.0028 (7)	0.0080 (7)	−0.0023 (6)
C7	0.0163 (8)	0.0130 (8)	0.0185 (8)	−0.0029 (6)	0.0075 (7)	−0.0016 (7)
C8	0.0142 (8)	0.0163 (8)	0.0162 (8)	−0.0005 (7)	0.0069 (7)	−0.0006 (6)
C9	0.0200 (9)	0.0151 (8)	0.0201 (9)	0.0005 (7)	0.0112 (7)	0.0011 (7)
C10	0.0195 (9)	0.0148 (8)	0.0199 (9)	0.0009 (7)	0.0115 (7)	−0.0007 (7)
C11	0.0155 (8)	0.0158 (8)	0.0150 (8)	0.0009 (6)	0.0061 (7)	0.0003 (7)
C12	0.0192 (9)	0.0152 (8)	0.0198 (9)	0.0020 (7)	0.0098 (7)	0.0039 (7)
C13	0.0179 (8)	0.0146 (8)	0.0185 (8)	0.0006 (6)	0.0089 (7)	−0.0017 (7)
C14	0.0194 (9)	0.0187 (8)	0.0181 (8)	−0.0002 (7)	0.0089 (7)	0.0014 (7)
C15	0.0205 (9)	0.0218 (9)	0.0202 (9)	0.0020 (7)	0.0100 (7)	0.0006 (7)
C16	0.0285 (10)	0.0260 (10)	0.0245 (9)	−0.0005 (8)	0.0157 (8)	−0.0008 (8)

### Geometric parameters (Å, º)

**Table:**

Br1—C2	1.9046 (17)	C10—C11	1.528 (2)
O1—C5	1.353 (2)	C10—H10A	0.9900
O1—H1	0.81 (2)	C10—H10B	0.9900
O2—C7	1.233 (2)	C11—C12	1.525 (2)
C1—C2	1.374 (2)	C11—C14	1.534 (2)
C1—C6	1.403 (2)	C11—H11	1.0000
C1—H1A	0.9500	C12—C13	1.525 (2)
C2—C3	1.398 (2)	C12—H12A	0.9900
C3—C4	1.379 (2)	C12—H12B	0.9900
C3—H3	0.9500	C13—H13A	0.9900
C4—C5	1.390 (2)	C13—H13B	0.9900
C4—H4	0.9500	C14—C15	1.522 (2)
C5—C6	1.414 (2)	C14—H14A	0.9900
C6—C7	1.484 (2)	C14—H14B	0.9900
C7—C8	1.516 (2)	C15—C16	1.530 (2)
C8—C9	1.531 (2)	C15—H15A	0.9900
C8—C13	1.543 (2)	C15—H15B	0.9900
C8—H8	1.0000	C16—H16A	0.9800
C9—C10	1.530 (2)	C16—H16B	0.9800
C9—H9A	0.9900	C16—H16C	0.9800
C9—H9B	0.9900		
			
C5—O1—H1	107.5 (19)	C9—C10—H10B	109.2
C2—C1—C6	120.60 (15)	H10A—C10—H10B	107.9
C2—C1—H1A	119.7	C12—C11—C10	109.65 (13)
C6—C1—H1A	119.7	C12—C11—C14	110.22 (13)
C1—C2—C3	120.96 (16)	C10—C11—C14	112.72 (13)
C1—C2—Br1	119.98 (13)	C12—C11—H11	108.0
C3—C2—Br1	119.03 (13)	C10—C11—H11	108.0
C4—C3—C2	119.17 (16)	C14—C11—H11	108.0
C4—C3—H3	120.4	C11—C12—C13	112.75 (13)
C2—C3—H3	120.4	C11—C12—H12A	109.0
C3—C4—C5	120.81 (16)	C13—C12—H12A	109.0
C3—C4—H4	119.6	C11—C12—H12B	109.0
C5—C4—H4	119.6	C13—C12—H12B	109.0
O1—C5—C4	117.44 (15)	H12A—C12—H12B	107.8
O1—C5—C6	122.35 (15)	C12—C13—C8	110.19 (13)
C4—C5—C6	120.21 (16)	C12—C13—H13A	109.6
C1—C6—C5	118.23 (15)	C8—C13—H13A	109.6
C1—C6—C7	122.23 (15)	C12—C13—H13B	109.6
C5—C6—C7	119.54 (15)	C8—C13—H13B	109.6
O2—C7—C6	119.47 (14)	H13A—C13—H13B	108.1
O2—C7—C8	119.83 (14)	C15—C14—C11	115.33 (14)
C6—C7—C8	120.70 (14)	C15—C14—H14A	108.4
C7—C8—C9	110.51 (13)	C11—C14—H14A	108.4
C7—C8—C13	110.65 (13)	C15—C14—H14B	108.4
C9—C8—C13	110.01 (13)	C11—C14—H14B	108.4
C7—C8—H8	108.5	H14A—C14—H14B	107.5
C9—C8—H8	108.5	C14—C15—C16	111.95 (14)
C13—C8—H8	108.5	C14—C15—H15A	109.2
C10—C9—C8	111.34 (13)	C16—C15—H15A	109.2
C10—C9—H9A	109.4	C14—C15—H15B	109.2
C8—C9—H9A	109.4	C16—C15—H15B	109.2
C10—C9—H9B	109.4	H15A—C15—H15B	107.9
C8—C9—H9B	109.4	C15—C16—H16A	109.5
H9A—C9—H9B	108.0	C15—C16—H16B	109.5
C11—C10—C9	112.15 (13)	H16A—C16—H16B	109.5
C11—C10—H10A	109.2	C15—C16—H16C	109.5
C9—C10—H10A	109.2	H16A—C16—H16C	109.5
C11—C10—H10B	109.2	H16B—C16—H16C	109.5
			
C6—C1—C2—C3	1.4 (3)	O2—C7—C8—C9	−27.0 (2)
C6—C1—C2—Br1	179.29 (12)	C6—C7—C8—C9	152.95 (14)
C1—C2—C3—C4	0.1 (3)	O2—C7—C8—C13	95.07 (18)
Br1—C2—C3—C4	−177.77 (13)	C6—C7—C8—C13	−84.95 (18)
C2—C3—C4—C5	−1.4 (3)	C7—C8—C9—C10	178.66 (13)
C3—C4—C5—O1	−178.60 (15)	C13—C8—C9—C10	56.18 (18)
C3—C4—C5—C6	1.2 (3)	C8—C9—C10—C11	−56.10 (18)
C2—C1—C6—C5	−1.6 (2)	C9—C10—C11—C12	54.50 (18)
C2—C1—C6—C7	178.51 (15)	C9—C10—C11—C14	177.69 (14)
O1—C5—C6—C1	−179.88 (15)	C10—C11—C12—C13	−55.68 (18)
C4—C5—C6—C1	0.3 (2)	C14—C11—C12—C13	179.67 (14)
O1—C5—C6—C7	0.0 (2)	C11—C12—C13—C8	57.25 (18)
C4—C5—C6—C7	−179.81 (15)	C7—C8—C13—C12	−178.73 (13)
C1—C6—C7—O2	−170.67 (15)	C9—C8—C13—C12	−56.33 (17)
C5—C6—C7—O2	9.5 (2)	C12—C11—C14—C15	−177.88 (14)
C1—C6—C7—C8	9.3 (2)	C10—C11—C14—C15	59.25 (19)
C5—C6—C7—C8	−170.52 (15)	C11—C14—C15—C16	172.51 (15)

### Hydrogen-bond geometry (Å, º)

**Table:**

*D*—H···*A*	*D*—H	H···*A*	*D*···*A*	*D*—H···*A*
O1—H1···O2	0.81 (2)	1.82 (2)	2.5527 (16)	148 (3)
C3—H3···O1^i^	0.95	2.59	3.483 (2)	157

Symmetry code: (i) −*x*+2, *y*−1/2, −*z*−1/2.
